# Refining the prediction of user satisfaction on chat-based AI applications with unsupervised filtering of rating text inconsistencies

**DOI:** 10.1098/rsos.241687

**Published:** 2025-02-05

**Authors:** Hae Sun Jung, Jang Hyun Kim, Haein Lee

**Affiliations:** ^1^ Department of Applied Artificial Intelligence, Sungkyunkwan University, Seoul 03063, Republic of Korea; ^2^ Department of Interaction Science, Sungkyunkwan University, Seoul 03063, Republic of Korea; ^3^ Department of Human–Artificial Intelligence Interaction, Sungkyunkwan University, Seoul 03063, Republic of Korea

**Keywords:** chat-based AI, sentiment analysis, natural language processing, user satisfaction, BERT

## Abstract

The swift development of artificial intelligence (AI) technology has triggered substantial changes, particularly evident in the emergence of chat-based services driven by large language models. With the increasing number of users utilizing these services, understanding and analysing user satisfaction becomes crucial for service improvement. While previous studies have explored leveraging online reviews as indicators of user satisfaction, efficiently collecting and analysing extensive datasets remain a challenge. This research aims to address this challenge by proposing a framework to handle extensive review datasets from the Google Play Store, employing natural language processing with machine learning techniques for sentiment analysis. Specifically, the authors collect review data of chat-based AI applications and perform filtering through majority voting of multiple unsupervised sentiment analyses. This framework is a proposed methodology for eliminating inconsistencies between ratings and contents. Subsequently, the authors conduct supervised sentiment analysis using various machine learning and deep learning algorithms. The experimental results confirm the effectiveness of the proposed approach showing improvement in prediction accuracy with cost efficiency. In summary, the findings of this study enhance the predictive performance of user satisfaction for improving service quality in chat-based AI applications and provide valuable insights for the advancement of next-generation chat-based AI services.

## 1. Introduction

The progress in artificial intelligence (AI) technology has led to significant transformations. In particular, chat-based services and applications that have emerged with the development of large-scale language models (LLMs) provide various services through interaction with users, deeply integrating into daily life. The user base of these chat-based AI services or applications continues to grow, underscoring the importance of analysing user satisfaction to provide improved services to users. User satisfaction is closely connected with service performance, and it is important to analyse it to improve service satisfaction [1–3]. To achieve this, researchers and industry practitioners endeavour to identify factors that positively influence service satisfaction by analysing user satisfaction.

According to previous research, online reviews can be employed as tools to analyse user experience or satisfaction [4–9]. However, the sheer volume of user feedback, such as the millions of reviews on platforms like the Google Play Store, presents a challenge for efficiently processing and analysing user satisfaction. Therefore, there is a necessity for research to efficiently handle extensive review datasets and derive valuable insights through the application of natural language processing (NLP) and machine learning methods, ensuring an accurate understanding of the sentiments and satisfaction levels presented in user feedback.

To address this, this study collects review data from chat-based AI applications available on the Google Play Store, incorporating both ratings and review text to predict user sentiment. At the initial stage, the dataset was divided into an 80:20 ratio. Eighty per cent was used for training and validation using fivefold cross-validation, while 20% was manually labelled and reserved as a separate test set for evaluation under real-world conditions. For the training data, rating-based sentiment labelling, which is a widely adopted method in existing research, was performed [2,7–9]. In this approach, reviews rated between 1 and 2 are labelled as negative, while those rated between 4 and 5 are labelled as positive. This method not only simplifies the interpretation of review data but also makes the sentiment labelling process relatively straightforward and intuitive. Moreover, it is well suited for handling large volumes of review data, making it particularly effective for machine learning and data analysis applications.

Afterwards, the authors employed majority voting methods using five unsupervised sentiment analysis techniques on the training dataset to filter out sarcasm or in cases where the chosen rating does not match the semantic context of the text. Subsequently, the training dataset was divided into two sets: (i) data labelled as positive or negative solely derived from ratings without applying unsupervised sentiment analysis and (ii) review data filtered through considering unsupervised sentiment analysis and ratings. Traditional machine learning models with embedding methods, as well as state-of-the-art (SOTA) deep learning models, such as bidirectional encoder representations from transformers (BERT), distilled BERT (DistilBERT) and robustly optimized BERT approach (RoBERTa), were then trained on each dataset in an attempt to determine the optimal method for predicting user satisfaction for the test dataset.

The key novelty of this study is its multi-method filtering approach. By employing a majority voting mechanism across five lexicon-based unsupervised sentiment analysis techniques, the authors assigned a filtering label to review data, ensuring that mismatches between text and ratings are addressed before model training. This approach offers two advantages.

— The first advantage is that each lexicon-based method analyses sentiment using different lexicons and their respective scores, resulting in clear distinctions. This approach compensates for the shortcomings of individual lexicons.
— The second advantage is the ability to maintain the fast processing speed of lexicon-based sentiment analysis while improving its relatively lower accuracy, thereby enhancing overall performance.

The experimental results demonstrated a notable enhancement in accuracy of approximately 2% across all models when utilizing the review data filtered through unsupervised sentiment analysis as a preliminary step. The findings demonstrate that this multi-method filtering is particularly useful in the context of chat-based AI application reviews, offering a more reliable way to analyse user feedback.

In summary, this research marks the first attempt within the domain of chat-based AI applications to enhance the predictive performance of user satisfaction by leveraging majority voting of unsupervised sentiment analysis methods as a preliminary filter. The authors anticipate that these research findings will offer valuable insights for enhancing service quality in chat-based applications and contribute to the advancement of next-generation chat-based AI services.

## 2. Literature review

### 2.1. Supervised and unsupervised sentiment analyses

Sentiment analysis retrieves emotions, opinions and attitudes from text data, assisting businesses in comprehending customer feedback from sources such as social media and reviews [10,11]. Specifically, sentiment analysis can be classified into two types: unsupervised and supervised sentiment analyses. Unsupervised sentiment analysis utilizes lexicon-based approaches to classify sentiment solely based on the provided text. In contrast, supervised learning-based sentiment analysis involves training models using pre-labelled sentiment data.

The lexicon-based approach utilizes sentiment dictionaries, which assign positive or negative labels to words based on their semantic orientation [12]. The characteristics of unsupervised sentiment analysis include its ability to be used without prior knowledge or labels of the data and its fast analysis speed. However, it is difficult to consider the context, leading to a lack of accuracy. On the contrary, machine learning models can be trained on text to perform supervised sentiment classification with labelled data. Supervised sentiment analysis is heavily influenced by the quantity and quality of the training data, enabling more accurate results. However, obtaining pre-labelled training data could demand considerable time and expense.

Research on sentiment analysis itself and its applications is being attempted in various fields. Araci [13] introduced a language model called Financial BERT (FinBERT) based on BERT specialized for sentiment analysis tasks in the financial domain, hypothesizing the existence of sentiment analysis models suitable for specific domains. The authors confirmed that FinBERT outperforms SOTA results for two financial sentiment analysis datasets and discovered that even fine-tuning with a small dataset outperforms SOTA machine learning methods. In [14], FinBERT was employed to derive sentiment scores from text data related to environmental, social and governance (ESG), which were subsequently integrated into stock price prediction models, resulting in improved prediction performance. Bonta *et al*. [15] pointed out that monitoring and summarizing reviews on the Web, given the extensive amount of text, is a challenging task, emphasizing the need for automated sentiment analysis. The authors classified movie reviews using sentiment analysis tools such as Natural Language Toolkit (NLTK), Textblob and the Valence Aware Dictionary and sEntiment Reasoner (VADER) and compared the performance of these tools. The authors found that VADER outperformed the other tools on the movie review dataset. Lee *et al*. [16] proposed a strategy for evaluating ESG using text-based automation technology. Specifically, the authors accurately classified ESG news into each category, followed by sentiment analysis using FinBERT. The authors aimed to devise a new equation through the computation of classification weights and sentiment analysis information to ensure the objectivity of ESG evaluation criteria. Jung *et al*. [17] conducted sentiment analysis on text related to Bitcoin from social media data, aiming to improve prediction performance by applying these sentiment analysis results to price prediction models. Ultimately, the authors demonstrated that unsupervised sentiment analysis on text data collected from Reddit, which represents investor sentiment, supports predicting Bitcoin price trends.

This study presents a novel approach by initially conducting multiple unsupervised sentiment analyses to combine the most frequent sentiment analysis results with review ratings for data filtering and subsequently employing supervised learning. The supposed framework combines the strengths of multiple unsupervised and supervised sentiment analyses.

### 2.2. Research on user satisfaction through online reviews and machine learning

Based on previous research, it is important to analyse service satisfaction since it is closely related to user satisfaction [1–3]. Prior research suggests that online reviews offer valuable insights into user satisfaction and may serve as a suitable method for assessment [4–6].

Lee *et al*. [1] proposed a framework employing machine learning models to predict user satisfaction in healthcare services using review data with ratings obtained from the Google Play Store. The authors utilized five machine learning techniques, and ultimately found that the combination of extreme gradient boosting (XGBoost) and term frequency–inverse document frequency (TF-IDF) showed the best performance in predicting service satisfaction for healthcare service users. Lee *et al*. [2] investigated the satisfaction of users of metaverse applications using VADER, along with various machine learning techniques and embeddings. The study demonstrated that the appropriate utilization of unsupervised and supervised sentiment analysis can extract more accurate information about user satisfaction. Lee *et al*. [3] conducted experiments by collecting review data with the search query ‘metaverse’ from YouTube and Google Play Store platforms. The authors found that the ensemble model combined with RoBERTa exhibited superior performance for sentiment analysis of metaverse service users. This empirically validates that employing deep learning and pre-trained language models for sentiment analysis holds the potential for enhancing user experiences within metaverse services. Hedegaard & Simonsen [4] investigated how usability and user experience (UX) information are distributed by analysing text data sourced from online reviews regarding software and video games. The study highlighted the importance of research to identify relevant dimensions of usability, as well as a deeper understanding of user concerns regarding various aspects of usability and UX from rich information reported online. Chen *et al*. [5] collected and analysed online comments on high-speed rail. The authors built and evaluated a passenger satisfaction evaluation framework based on the analysis results. Ultimately, the authors highlighted that online reviews can be utilized for predicting user satisfaction and improving the quality of service. Yu [6] aimed to overcome the limitations of survey-based user satisfaction assessments in the existing literature and to explore perceptions from a broader perspective by utilizing YouTube online reviews to comprehend the public’s overall outlook on robots functioning as frontline staff in the hotel industry.

In summary, the literature review emphasizes the importance of automatically analysing large volumes of online review texts through machine learning to understand and enhance user satisfaction, ultimately revealing its potential to stimulate the success of services or products.

## 3. Material and methods

**Figure 1.**
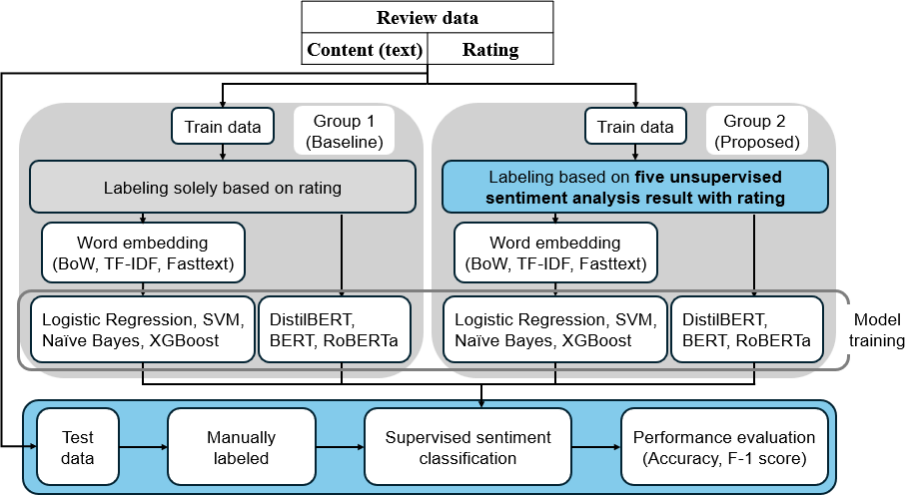
Experimental outline.

The proposed framework has the following experimental outline (figure 1).

### 3.1. Data collection and preprocessing

The authors aimed to gather a diverse range of application review data for chat-based AI from the Google Play Store. For this purpose, the authors selected applications named ‘Replica’, ‘Bing’, ‘ChatGPT’ and ‘POE’. Using a Python scraper with google-play-scraper, the authors collected English data, resulting in a total of 278 556 review data comprising ratings and review contents. Subsequently, the authors performed stop-word removal and lemmatization. Additionally, as texts that are too short often lack meaningful content, the authors removed data shorter than 20 characters, resulting in 179 967 remaining data.

Following prior research, ratings were used to label the collected data for binary classification [2,7–9]. Specifically, ratings of 1−2 were labelled as negative (0), and ratings of 4−5 were labelled as positive (1). Data with a rating of 3 were eliminated due to their potential to function as outliers in both positive and negative categories. The specific reasons are as follows. First, the authors aimed to focus on clear sentiment polarity. Our main goal was to classify user satisfaction into distinct positive and negative categories. A 1–5 scale can introduce ambiguity in the middle range (e.g. 3 out of 5), making it difficult to clearly define the sentiment as purely positive or negative. By using binary labels, the authors ensured a more direct and interpretable analysis of user satisfaction. Second, the authors sought to achieve improved model performance. Through extensive prior research, the authors found that using binary labels produced more stable and accurate results across the various machine learning models employed [18–21]. Introducing a broader sentiment spectrum led to challenges in consistency and predictive accuracy due to noise and ambiguity in mid-range ratings. Additionally, in filtering through the five unsupervised learning models, each model uses different polarity ranges. If this study were to use middle-range values, the authors would have had to unify the polarity range of those middle values arbitrarily, which could introduce bias. To avoid this, the authors chose to exclude the middle-range label (i.e. the 3-star rating). The third reason is the relevance to user satisfaction. Ultimately, this research focused on understanding and predicting user satisfaction, which was best captured through clear positive or negative experiences. While a 1–5 scale offers more detail, it interrupts clarity in the decision-making process that companies use to either improve or resolve issues related to user satisfaction based on feedback. For these reasons, the authors chose to use binary labels of positive and negative.

**Figure 2.**
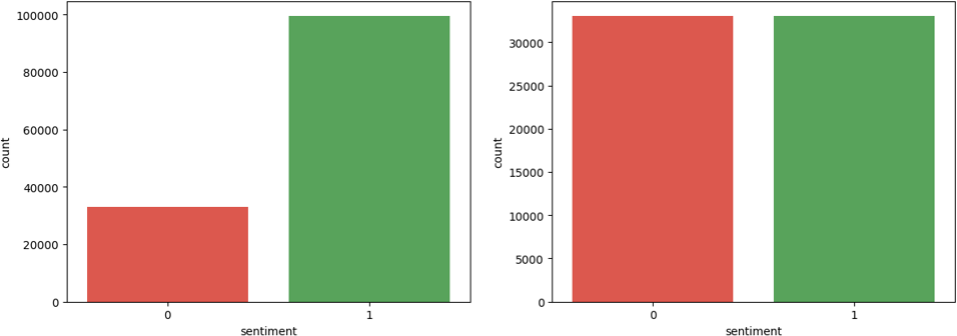
Labelled training dataset solely based on ratings before undersampling (left) and after undersampling (right).

After this exclusion, there were 165 760 data remaining. Among these data, 33 152 data points were randomly selected for the test dataset, leaving 132 608 data for the training dataset. The label from the rating revealed that the training dataset contained 99 550 positive data and 33 058 negative data (figure 2, left). Subsequently, undersampling was performed based on the number of negative review data to balance the dataset with 33 058 positive and 33 058 negative data (figure 2, right).

However, according to previous studies, sentiments expressed in online reviews may not always align with ratings, and there are two possible scenarios [2]. The first case is when a written review clearly expresses a positive sentiment but gives a low rating. The other case is when a written review carries a negative sentiment but the reviewer awards a high rating (table 1). These unusual cases during the labelling process could potentially adversely affect the supervised learning-based sentiment classifier to be trained.

**Table 1.** Examples of data where ratings and text are inconsistent.

index	contents	rating	sentiment_label (based on rating)	date
1	I 😄doing no problem in the Bing app safety to use the controlled for me	1	negative	1 January 2020 09.58.33
2	it’s nice 💯	1	negative	19 December 2023 18.12.07
3	difficult subscribing	5	positive	7 December 2023 18.21.42
4	slow loading	4	positive	2 July 2023 10.38.51
5	very helpful microsoft bing 😘	2	negative	1 March 2024 01.27.27

### 3.2. Unsupervised sentiment analysis methods

To address these cases of inconsistency between text and ratings, the authors initially utilized five unsupervised learning-based sentiment analysis modules for filtering and aimed to enhance the performance of the supervised sentiment analysis.

The unsupervised sentiment analysis utilizes a lexicon-based approach, relying on lexicons to quantify the positivity or negativity of words and analyse sentiment in data without specified labels [12,15]. Researchers initially organize sentiment word lists through manual, lexical and corpus-based approaches to construct sentiment lexicons. Subsequently, polarity scores of given reviews are determined based on identified positive and negative indicators within these lexicons. Each unsupervised sentiment analysis method can return different results depending on the sentiment lexicon utilized. Therefore, the authors aimed to utilize various unsupervised sentiment analysis libraries to obtain optimal results. The authors employed Textblob, Afinn, VADER, Pattern and Sentiwordnet from Python for lexicon-based unsupervised sentiment analysis [22–26]. Each positive and negative threshold was set through exploratory analysis to classify more towards the negative sentiment based on the inherent skewness towards positivity in both the data and the lexicon itself.

#### 3.2.1. Textblob

Textblob provides access to NLP tasks through an application programming interface (API) such as tokenization and sentiment analysis [22]. It is a rule-based approach relying on simple lexicons from NLTK and polarity scores, evaluating sentiment at the sentence level. The Textblob sentiment classifier returns two attributes on the given input sentence: polarity and subjectivity, both of which range between −1 and 1. Polarity indicates whether the sentiment is positive or negative, with 0 representing neutrality. Subjectivity illustrates whether the sentence is objective or subjective, with 0 being objective and 1 being subjective. Textblob disregards unknown words and calculates the final score by evaluating words and phrases capable of polarity assignment, subsequently averaging them. For sentiment analysis, the authors only employed polarity. If the polarity value is greater than 0.1, it is labelled as positive. Conversely, if the value is less than 0.1, it is labelled as negative, and if it equals 0, it is labelled as neutral since it may signify that the target words are not included in the lexicon:


Positive: Polarity>0.1

Neutral: Polarity=0.0

Negative: Polarity≤0.1 and Polarity !=0.

#### 3.2.2. Afinn

Afinn is a lexicon-based sentiment analysis module developed by Finn Årup Nielsen, which is based on a word list known as AFINN-111 [23]. It can be used in various applications such as simple text mining tasks or social media analysis. Sentiment scores can be computed using the score function of afinn and evaluated at the word level without considering context, Afinn, which returns an integer value. Following a similar approach, a sentiment score value greater than 2 was categorized as positive, while a value below 2 was assigned a negative label; a value of 0 denoted neutral:


$$Positive:\;Sentiment_{score}>2.0$$

$$Neutral:\;Sentiment_{score}=0.0$$

$$Negative:\;Sentiment_{score}\leq 2.0\;and\;Sentiment_{score}\;!=0.$$

#### 3.2.3. VADER

VADER is typically used in conjunction with a lexicon of sentiment-related features, which are words or phrases that are labelled as positive or negative based on their semantic orientation [24]. VADER is particularly optimized for social media data, as it integrates lexicons that account for capitalization, punctuation and emojis, making it suitable for informal language. Its fast processing speed enables efficient handling of extensive text data and provides more refined analysis by incorporating intensity like adverbs and emojis in sentiment scoring. The polarity_scores() function can be used to find polarity scores for a given sentence, returning metrics for negative, neutral, positive and compound sentiment. The compound score is computed by normalizing the sum of all lexicon ratings to a range between −1 and +1. This is useful for setting standardized thresholds for categorizing sentences as positive, neutral or negative. In the case of VADER, the authors followed the commonly used threshold [25]:


$$Positive:\;Compound_{score}>0.1$$

$$Neutral:\;-0.1<Compound_{score}\leq 0.1$$

$$Negative:\;Compound_{score}\leq -0.1.$$

#### 3.2.4. Pattern

Pattern.en is a rapid, shallow parser for the English language based on regular expressions, which identifies components of sentences (e.g. nouns, verbs) through a finite state part-of-speech (POS) tagger [26]. It uses its own lexicon to evaluate word sentiment, providing basic sentiment analysis with a focus on Web scraping and data mining. The sentiment() function within pattern.en provides both the sentiment score and confidence level for a given text. The sentiment score indicates the sentiment intensity of the given text. This value typically ranges from −1 to 1, with higher values representing more positive sentiment. In addition, the confidence level is a measure of how reliable the sentiment score is. It is usually expressed as a value between 0 and 1, where higher values mean the sentiment score is more trustworthy. Employing a similar methodology, a sentiment score exceeding 0.1 was classified as positive, whereas a score below or equal to 0.1 was labelled as negative; a score of 0 indicated neutrality:


$$Positive:\;Sentiment_{Score}>0.1$$

$$Neutral:\;Sentiment_{Score}=0.0$$

$$Negative:\;Sentiment_{Score}\leq 0.1\;and\;Sentiment_{Score}\;!=0.$$

#### 3.2.5. SentiWordNet

SentiWordNet is an unsupervised sentiment analysis tool included in the NLTK module which consists of 147 306 sets of synonyms annotated with three sentiment scores: positive, negative and objective [15,27]. Based on the WordNet lexical database, SentiWordNet offers detailed sentiment analysis with scores based on word meanings, making it useful for context-sensitive sentiment evaluation. Sentiment scores in NLTK are calculated from the polarity scores of each Sysnet in WordNet, with each Sysnet’s three sentiment scores ranging from 0.0 to 1.0. Sysnets in WordNet are uniquely identified by a POS and ID pair, with the Sysnet term column listing the included terms and their sentiment scores (see table 2).

**Table 2.** Example of data from SentiWordNet.

word	POS tagging	positive_score	negative_score
sad	a	0.125	0.75
	s	0.0	0.25
happy	a	0.875	0.0
	s	0.125	0.0

The sentiment score of the target review data was computed by adding up all the term sentiment scores as shown below:


$$sentiment_{score}=\;\sum_{i=1}^{m}term_{score}\left(P_{i}\right)-\sum_{j=1}^{n}term_{score}\left(N_{j}\right),$$

where $P_{i}$ represents the ith positive word in the review and $N_{j}$ represents the jth negative word in the review. The sentiment classification threshold of SentiWordNet was also set to 0.

Ultimately, the authors conducted sentiment analysis using five unsupervised sentiment analysis modules and derived the results based on the sentiment that appeared most frequently among these five modules. This process aimed to achieve optimal unsupervised sentiment analysis results by considering the strengths and weaknesses of the lexicons used by each library, ultimately obtaining refined data free of sarcasm and performing better in supervised sentiment classification. Furthermore, this process also allows for the extraction of neutral sentiments as results, thus enhancing the possibility of conducting a more detailed sentiment analysis.

**Figure 3.**
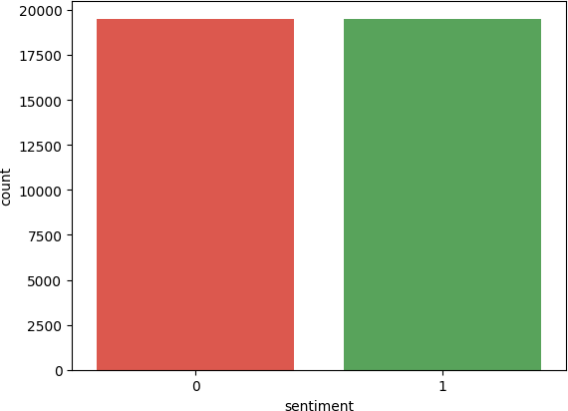
Labelled training dataset based on ratings with unsupervised sentiment analysis results after undersampling.

After employing unsupervised sentiment analysis libraries, only the data where the unsupervised learning results matched the ratings were retained (table 3). The obtained sentiment results were 27 635 positive data (1) and 19 517 negative data (0). For the training phase, a second round of undersampling was conducted, resulting in 19 517 positive and 19 517 negative unsupervised sentiment analysis filtered data (figure 3).

**Table 3.** Example of data after initial filtering using five unsupervised sentiment analyses.

content	sentiment (rating)	Textblob	Afinn	VADER	Pattern	SentiWordNet	sentiment (filtered)
best free GPT-4 app	1	1	1	1	1	1	1
use less app it does not create anything with AI	0	0	1	0	0	0	0
it is fake	0	0	neutral	0	neutral	0	0
it is the wonderful app I have ever seen in my...	1	1	1	1	1	1	1
last update ruined Replika. Hopefully it gets ...	0	1	0	0	1	0	0

#### 3.2.6. Statistical analysis between labels attained through unsupervised sentiment analysis

The authors conducted additional statistical analyses to determine whether there are significant differences among the labels from five unsupervised sentiment analysis methodologies. First, the authors performed the Friedman test, which is a non-parametric approach that compares three or more related samples [28]. This test assesses whether the differences among groups are significant. Subsequently, the authors implemented *post hoc* tests to identify which methodologies are significantly different from each other. For the *post hoc* analysis, Dunn’s test was employed [29]. This test is used in conjunction with the Friedman test to compare the mean differences among multiple related groups.

According to the results of the statistical tests, the Friedman test indicated significant differences, with a Friedman test statistic of 20 516.67 and a *p*-value less than 0.00. The results of Dunn’s test showed that if the *p*-value is less than 0.05, there is a statistically significant difference between the two methodologies. Table 4 presents the results of Dunn’s test. Based on these results, the authors confirmed the existence of distinct differences among the methodologies. Therefore, integrating all five methodologies provides a less biased approach compared with relying on a single methodology.

**Table 4.** Pairwise comparison results from Dunn’s test for sentiment analysis.

	Textblob	Afinn	VADER	Pattern	SentiWordNet
Textblob	1	*p* < 0.00	3.13 × 10^−89^	2.49 × 10^−106^	*p* < 0.00
Afinn	*p* < 0.00	1	*p* < 0.00	*p* < 0.00	*p* < 0.00
VADER	3.13 × 10^−89^	*p* < 0.00	1	6.23 × 10^−1^	2.97 × 10^−114^
Pattern	2.49 × 10^−106^	*p* < 0 .00	6.23 × 10^−1^	1	1.69 × 10^−96^
SentiWordNet	*p* < 0 .00	*p* < 0.00	2.97 × 10^−114^	1.69 × 10^−96^	1

### 3.3. Word embedding methodologies

Word embedding is employed in NLP tasks to convert words into fixed length numerical vectors [30,31]. These vector representations capture the semantic similarity between words and enable computational manipulation, allowing computers to process text interpretation. Specifically, words with similar meanings are represented by similar vectors and are located close to each other. This helps in comprehending the semantic relationships between words and interpreting the meaning of sentences in NLP tasks. Word embedding is primarily generated during the training of neural networks or machine learning-based language models and is essential for obtaining efficient and accurate results.

#### 3.3.1. Bag of words

The bag of words (BoW) method represents each word in a document as a vector by recording the frequency of occurrence of each term [32]. Although there is no equation in the BoW model, it undergoes a series of steps to represent the occurrence frequency of each term. First, it identifies the types of words appearing in all documents and assigns a unique index to each word. Then, it converts each document into a vector representing the occurrence frequency of those terms. In this process, the position corresponding to the index of each word contains the frequency of occurrence of that word. These vectors are then collected to create a document-term matrix.

#### 3.3.2. Term frequency–inverse document frequency

TF-IDF is a statistical method employed to calculate the importance of words [33]. TF-IDF helps determine how important a specific term is in a given document. TF represents the frequency of a specific term appearing in a document, computed by dividing the occurrences of the word by the total word count of the document. On the other hand, IDF is a metric that indicates the scarcity of a specific word across all documents. IDF is calculated as the logarithm of the inverse of the frequency of occurrence of a particular word. TF-IDF is calculated by multiplying these two metrics together:


$$TF\text{-}IDF\left(t,d,D\right)=TF\left(t,d\right)\times IDF\left(t,D\right)=\frac{f_{t,d}}{max_{t^{\prime }\in d}f_{t^{\prime },d}}\times \log\left(\frac{N}{\left|\left\{d\in D:t\in d\right\}\right|}\right).$$

In the equations, for TF(t,d), t represents the word, *d* represents the document, $f_{t,d}$ is the frequency of the word t appearing in document *d* and $max_{t^{\prime }\in d}f_{t^{\prime },d}$ is the highest frequency of any term within the document *d*. For IDF(t,D), *D* stands for the total quantity of documents and $\left|\left\{d\in D:t\in d\right\}\right|$ represents the quantity of documents that include the word t.

#### 3.3.3. FastText

FastText is a word embedding based on the Skip-gram model used in Word2Vec [34,35]. The core idea of fastText is to divide words into character-level n-grams for processing. FastText utilizes these n-gram representations to understand the meaning of words and learn embedding vectors. This enables robust performance even for rare or misspelled words. In the case of fastText, the method of calculating scores may differ, but the objective function is the same as that of the Skip-gram model with negative sampling. The objective function of the Skip-gram model with negative sampling can be formulated as follows:


$$\log P\left(w_{O}|w_{I}\right)+\sum_{i=1}^{k}E_{w_{i}∼P_{n}\left(w\right)}\left[\log P\left(w_{i}|w_{I}\right)\right],$$

where $w_{I}$ is the centre word, $w_{O}$ is the context word (i.e. surrounding word), $P(w_{O}|w_{I})$ denotes the probability that the context word $w_{O}$ occurs given the centre word $w_{I}$, $P_{n}(w)$ represents the probability of word w sampled from the noise distribution, *k* denotes the quantity of negative samples sampled for each training instance and $P(w_{i}|w_{I})$ indicates the probability that the negative sample $w_{i}$ occurs given the centre word $w_{I}$.

### 3.4. Supervised machine learning models

In this part, the authors describe algorithms utilized for supervised learning of binary sentiment classification using embeddings for labelled data from both (i) solely based on ratings and (ii) based on ratings combined with unsupervised sentiment analysis results, along with BERT, DistilBERT and RoBERTa embeddings paired with classifiers.

#### 3.4.1. Logistic regression

Logistic regression is a model used to predict the probability of an event by combining independent variables linearly [36]. Utilizing logistic regression for binary classification is straightforward yet demonstrates effectiveness across various tasks. The hypothesis of logistic regression is as follows:


$$h_{\theta }\left(x\right)=g\left(z\right)=\frac{1}{1+e^{-W^{T}x}},$$

where $h_{\theta }\left(x\right)$ is the probability of the predicted class for input data *x*, while $W^{T}x$ is the sum of the weighted features and bias of the input. These probabilities are obtained through the sigmoid function g(z), which converts them into probability values between 0 and 1. Then, predictions are classified as 0 or 1 based on these probability values. Typically, values equal to or greater than 0.5 are categorized as 1, while those less than 0.5 are assigned to 0.

#### 3.4.2. Support vector machine

The support vector machine (SVM) is designed to determine the hyperplane for classification [37]. The hyperplane maximizes the margin, which signifies the distance between the hyperplane and the support vectors. Once the decision boundary is defined, classification is performed by determining which side of the boundary a new input falls on. Classification employing SVM is robust and resistant to overfitting. Nevertheless, it requires testing for various parameter combinations. The formula for the margin of the support vectors is as follows:


$$margin=\;\frac{2}{\left\|w\right\|},$$

where w is the weight of the hyperplane.

#### 3.4.3. Naive Bayes

Naive Bayes classifier is a probabilistic machine learning method that applies Bayesian theory, assuming that each dimension of the input variables is conditionally independent [38]. The classifier calculates the probability of a given sample belonging to each class based on the observed features and then assigns the sample to the class with the highest probability. The naive Bayes classifier performs effectively in numerous intricate real-world situations and is utilized for classification tasks in various domains despite the simple assumption and design. Bayesian theory can be expressed as follows:


$$P\left(H|e\right)=\frac{P\left(e|H\right)P\left(H\right)}{P\left(e\right)}.$$

$P\left(H|e\right)$ represents the posterior probability of the class given the predictor, $P\left(e|H\right)$ is the likelihood indicating the probability of the predictor given the class, $P\left(H\right)$ stands for the prior probability of the class and P(e) denotes the evidence.

#### 3.4.4. Extreme gradient boosting

XGBoost employs an ensemble approach by combining several weak decision trees [39]. It assigns weights to the errors of these weak prediction models and progressively integrates them into subsequent models, thereby establishing a strong predictive model. Due to parallel processing, XGBoost offers rapid learning and classification speeds, but without proper parameter tuning, overfitting can occur easily. XGBoost seeks to minimize the following objective function:


$$L\left(\phi \right)=\sum_{i}l\left({\hat{y}}_{i},\;y_{i}\right)+\sum_{k}\Omega \left(f_{k}\right),$$

where l is the loss function between ${\hat{y}}_{i}$ and $y_{i}$ and Ω denotes the regularization parameter.

#### 3.4.5. Bidirectional encoder representations from transformers

BERT stands as a SOTA transformer architecture adapted for NLP tasks. BERT utilizes a bidirectional transformer encoder to consider the bidirectional context, enabling it to encode a given word by considering both preceding and following words [40]. Pre-trained on an extensive text corpus, BERT captures contextualized representations, providing a robust foundation for downstream tasks. The main loss functions used in BERT pre-training are primarily the masked language model (MLM) loss and the next sentence prediction (NSP) loss. The MLM loss involves masking certain words within the input sentences and prompting the model to predict these masked words. The model generates embeddings for the masked words and computes the loss by comparing them with the actual answers. The NSP loss considers that BERT receives sentence pairs as input during pre-training. This loss function prompts the model to predict whether the second sentence immediately follows the first sentence. The model generates embeddings for both sentences, learns the relationship between sentence pairs and performs the next sentence prediction based on this information. One of the advantages of BERT is its capability for fine-tuning downstream tasks [41]. During fine-tuning, task-specific layers are added to adapt to particular tasks. This approach has achieved high success across various NLP benchmarks.

#### 3.4.6. Distilled BERT

DistilBERT is a lightweight version of BERT, based on the main ideas of BERT but reduced in model size and computational cost while preserving BERT’s performance [42]. To achieve this, DistilBERT utilizes the following key techniques.

First, DistilBERT retains the overall architecture of BERT but reduces the number of layers by half and removes the pooler and token-type embeddings. The authors prioritized reducing the number of layers, as reducing the hidden state dimension of the transformer does not have a significant impact. Additionally, DistilBERT utilizes transfer learning with pre-trained weights from BERT and adopts training techniques similar to RoBERTa, including (i) dynamic masking and (ii) excluding NSP. DistilBERT reduces the size of BERT’s model by 40% and achieves a 60% increase in speed, while maintaining performance at around 97%, with only a minimal decrease.

#### 3.4.7. Robustly optimized BERT approach

RoBERTa is an improved model of the BERT architecture developed by the Facebook AI research team in 2019 [43]. Compared with BERT, RoBERTa has the following key features and improvements.

First, RoBERTa introduced a more flexible and efficient dynamic masking strategy by enhancing the fixed masking strategy used in BERT. This helps the model effectively learn more information from pre-training data. Second, RoBERTa was pre-trained using a larger scale model and a more extensive corpus compared with BERT, which has contributed to enhancing the model’s language understanding capabilities. With these changes, RoBERTa demonstrates SOTA performance across various NLP benchmarks [44].

## 4. Results

### 4.1. Experimental details

The experiments were conducted using the following hardware specifications: Intel Core i7-10700F CPU, 32 GB RAM and an NVIDIA GeForce 3080 GPU. These specifications provided the necessary computational power for efficiently training and evaluating the models. Additionally, the following versions of Python libraries were utilized in the experiments: Scikit-learn v. 1.5.2, XGBoost v. 1.7.5, Gensim v. 4.2.0, Torch v. 1.12.0 and Transformers v. 4.26.1.

For all models, hyperparameters were optimized through a grid search combined with fivefold cross-validation. This approach ensured that the models are trained and validated on different subsets of the data, which helps in mitigating overfitting and provides a more robust evaluation of model performance. The best-performing model was then selected based on these evaluations and used to derive results on the test set. This rigorous methodology allows for a thorough assessment of the models’ capabilities in sentiment analysis tasks.

### 4.2. Evaluation metrics

The most frequently employed metrics for evaluating performance in classification problems are accuracy and F-1 score [45]. Accuracy indicates the proportion of accurately classified instances out of the total samples, while the F-1 score represents the harmonic mean of the model’s precision and recall. In binary classification, accuracy is crucial for assessing model performance. However, when dealing with class imbalances, relying solely on accuracy can be problematic. In such scenarios, the F-1 score becomes invaluable. It provides a comprehensive evaluation of the model’s performance and offers a more reliable measure, especially considering class imbalances. Therefore, the authors employed these two evaluation metrics for the performance evaluation.

### 4.3. Results of supervised sentiment analysis

The classification performance evaluation for the manually labelled test dataset of supervised sentiment analysis trained on data labelled with and without majority voting filtering is described in this section.

Table 5 presents the comparison of supervised sentiment analysis performance using BoW embeddings with machine learning on the test dataset, both with and without filtering. Among this approach, the SVM with filtering demonstrated the best performance. Additionally, it was observed that the presence of filtering generally led to a performance improvement of approximately 1−1.5% across the models.

**Table 5.** Comparison of supervised sentiment analysis performance employing BoW embeddings.

model	class		evaluation metric	without filtering	with filtering
logistic regression	30 662		accuracy	0.8808	0.8953
	pos	21 820	F-1 score	0.9165	0.9269
	neg	8842		0.7917	0.8155
SVM	30 662		accuracy	0.8821	0.8984
	pos	21 820	F-1 score	0.9175	0.9293
	neg	8842		0.7934	0.8198
naive Bayes	30 662		accuracy	0.8678	0.8780
	pos	21 820	F-1 score	0.9065	0.9140
	neg	8842		0.7743	0.7899
XGBoost	30 662		accuracy	0.8813	0.8962
	pos	21 820	F-1 score	0.9166	0.9274
	neg	8842		0.7942	0.8181

Subsequently, table 6 presents a comparison of supervised sentiment analysis performance using TF-IDF embeddings on the test dataset. In this approach, the SVM model with filtering achieved the best performance. Moreover, it was observed that applying filtering generally resulted in a performance improvement of approximately 1−1.5% across all models.

**Table 6.** Comparison of supervised sentiment analysis performance employing TF-IDF embeddings.

model	class		evaluation metric	without filtering	with filtering
logistic regression	30 662		accuracy	0.8790	0.8951
	pos	21 820	F-1 score	0.9148	0.9266
	neg	8842		0.7917	0.8168
SVM	30 662		accuracy	0.8792	0.8953
	pos	21 820	F-1 score	0.9148	0.9267
	neg	8842		0.7925	0.8170
naive Bayes	30 662		accuracy	0.8578	0.8715
	pos	21 820	F-1 score	0.8985	0.9091
	neg	8842		0.7631	0.7810
XGBoost	30 662		accuracy	0.6106	0.6235
	pos	21 820	F-1 score	0.6406	0.6617
	neg	8842		0.5751	0.5756

Table 7 provides a comparison of supervised sentiment analysis performance using fastText embeddings on the test dataset. In this case, the XGBoost model with filtering delivered the highest performance. Additionally, applying filtering consistently led to a performance improvement of approximately 1% across all models.

**Table 7.** Comparison of supervised sentiment analysis performance employing fastText embeddings.

model	class		evaluation metric	without filtering	with filtering
logistic regression	30 662		accuracy	0.8670	0.8788
	pos	21 820	F-1 score	0.9049	0.9142
	neg	8842		0.7790	0.7941
SVM	30 662		accuracy	0.8660	0.8775
	pos	21 820	F-1 score	0.9039	0.9131
	neg	8842		0.7785	0.7926
naive Bayes	30 662		accuracy	0.8149	0.8213
	pos	21 820	F1-score	0.8642	0.8667
	neg	8842		0.7092	0.7287
XGBoost	30 662		accuracy	0.8697	0.8793
	pos	21 820	F-1 score	0.9071	0.9145
	neg	8842		0.7820	0.7947

Table 8 provides a comparison of supervised sentiment analysis performance using transformer-based models on the test dataset. The DistillBERT model with filtering achieved the highest F-1 score for the positive class, while BERT with filtering delivered the best performance in terms of accuracy and F-1 score for the negative class.

**Table 8.** Comparison of supervised sentiment analysis performance employing transformer-based models.

model	class		evaluation metric	without filtering	with filtering
DistilBERT	30 662		accuracy	0.8969	0.9179
	pos	21 820	F-1 score	0.9288	0.9436
	neg	8842		0.8133	0.8487
BERT	30 662		accuracy	0.8973	0.9183
	pos	21 820	F-1 score	0.9284	0.9433
	neg	8842		0.8181	0.8538
RoBERTa	30 662		accuracy	0.8935	0.9122
	pos	21 820	F-1 score	0.9269	0.9401
	neg	8842		0.8041	0.8362

**Figure 4.**
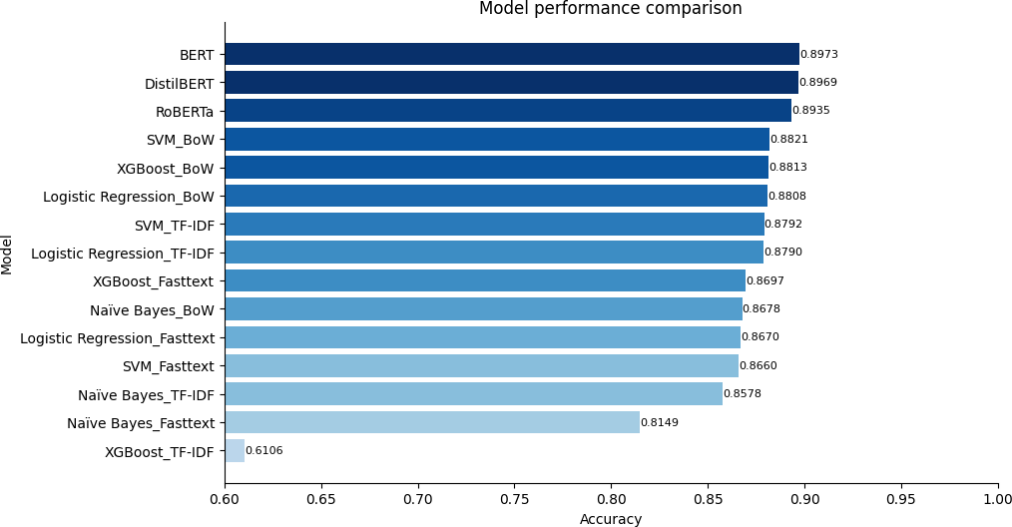
The supervised sentiment analysis performance on the test dataset trained on labelled data without filtering.

**Figure 5.**
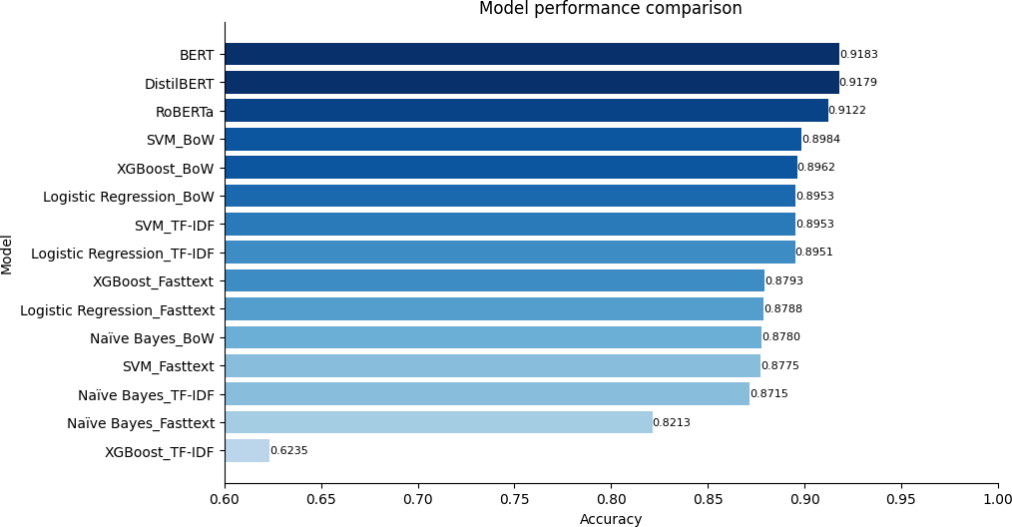
The supervised sentiment analysis performance on the test dataset trained on labelled data with filtering.

Overall, transformer-based methods significantly outperformed traditional machine learning techniques combined with embeddings (figures 4 and 5). Notably, among the embedding methods, BoW showed the best performance, followed closely by TF-IDF, which also demonstrated strong results. However, fastText did not perform as well in comparison.

In summary, the highest accuracy of supervised binary sentiment classification for data filtered through unsupervised sentiment analysis was 91.83%, with the lowest performance at 62.35%. On the other hand, for the unfiltered data, the highest performance was 89.73%, with the lowest at 61.06%. There was an approximately 2.1% performance difference in the case of the highest performance, and an around 1.3% difference for the lowest performance. The significant gap in sentiment classification performance validates the effectiveness of the proposed approach in this research, which involves utilizing the most frequent sentiment after employing multiple unsupervised sentiment analyses along with ratings for initial filtering, followed by supervised sentiment analysis.

## 5. Conclusion

The industry for chat-based AI services related to LLM is rapidly evolving, driven by advancements in LLM and AI technologies, as well as improvements in computing power. Taking this trend into consideration, various domains employing chat-based AI endeavour to improve user satisfaction. To understand and improve user satisfaction, relevant review data can be utilized [1–9,46]. In this study, the authors collected review data of chat-based AI applications consisting of ratings and text, for understanding the satisfaction of service users more accurately.

However, the star ratings provided by users do not directly correlate with the content of their reviews [2]. This study aimed to address this problem by analysing the sentiment of user reviews and addressing instances of inconsistency between text and ratings to enhance the performance of sentiment analysis models. In detail, initial filtering was conducted through five unsupervised sentiment analysis modules, followed by supervised sentiment analysis. This process was significant in deriving optimal unsupervised learning results by considering the strengths and weaknesses of each lexicon in each unsupervised sentiment analysis library. Moreover, even neutral sentiments could be identified and extracted, enabling more detailed sentiment analysis. Subsequently, traditional machine learning models and embedding methods, as well as SOTA deep learning models such as BERT, DistilBERT and RoBERTa, were trained on both the review data filtered through unsupervised learning and data labelled based solely on ratings. On the manually labelled test data, supervised models trained on data filtered through unsupervised sentiment analysis showed an improvement in accuracy of approximately 2% compared with data labelled based on ratings.

The practical implications of this study are notable. Based on the results, models based on BERT showed better performance in both models with and without text inconsistency compared with machine learning-based models and embedding combinations. These results can be utilized for developing cost-efficient automated online review analysis systems in the chat-based AI domain. Additionally, BoW was shown to be more appropriate for representing words in review data compared with TF-IDF or fastText. Therefore, from the perspective of companies with limited resources, utilizing BoW when constructing sentiment classifiers is advantageous. Furthermore, the proposed framework can be utilized by product or service providers to gain accurate insights for users even in cases where there are only texts without ratings. Through the proposed methodology, it is possible to initially filter extensive text review data and perform a more detailed analysis of the extracted aggregated data.

Another implication of this study is that LLMs were not considered as classification models. While LLMs require high-performance hardware and large-scale datasets, this study was designed to enable experimentation even in resource-constrained environments. This aspect is a critical factor when considering the applicability of models in real-world service environments with limited resources. Consequently, the result of this research is expected to provide crucial insights for enhancing service quality in chat-based AI applications and contribute to the improvement of next-generation chat-based AI services.

There are several limitations that need to be handled. First, this research only analysed review data in English. Future research could validate across various languages. Additionally, the experiments were conducted without significantly adjusting the threshold of unsupervised sentiment analysis. It will be beneficial to conduct more experimental explorations in future research, and adjusting the threshold more exquisitely could lead to more accurate supervised sentiment analysis results.

## Data Availability

Data and relevant code for this research work are stored in GitHub: https://github.com/HaeSunJung/Chat_based_LM and have been archived within the Zenodo repository [47].
